# Macrophages in tumors with downregulated MHC-I expression: emerging therapeutic opportunities and translational challenges

**DOI:** 10.1093/cei/uxag047

**Published:** 2026-08-11

**Authors:** Adrianna Piatakova, Michal Smahel

**Affiliations:** Department of Genetics and Microbiology, Faculty of Science, Charles University, BIOCEV, Vestec, Czech Republic; Department of Genetics and Microbiology, Faculty of Science, Charles University, BIOCEV, Vestec, Czech Republic

**Keywords:** tumor-associated macrophages, MHC class I, antigen presentation, phagocytosis, single-cell sequencing, β2-microglobulin, tumor microenvironment

## Abstract

Tumor-associated macrophages (TAMs) are the dominant component of the tumor microenvironment and exhibit remarkable phenotypic plasticity and heterogeneity that extends beyond the classical M1/M2 polarization, as revealed by single-cell transcriptomics across multiple cancer types. Downregulation of major histocompatibility complex class I (MHC-I) on tumor cells is a common immune evasion strategy that profoundly shapes TAM composition and function. Conversely, TAMs reciprocally modulate tumor MHC-I expression. This review provides an overview of the verified and hypothetical mechanisms of bidirectional regulatory interactions between MHC-I on tumor cells and TAMs. Since these interactions likely differ between tumors with reversible and irreversible MHC-I downregulation, their potential significance in tumor immunotherapy should be assessed separately for each MHC-I reduction mechanism.

## Teaser

Bidirectional regulatory interactions between tumor-associated macrophages (TAMs) and major histocompatibility complex class I (MHC-I) molecules on tumor cells shape the tumor microenvironment and immunotherapy responses. MHC-I downregulation alters TAM composition and function, including phagocytosis. Repolarizing TAMs and activating phagocytosis represent promising therapeutic strategies for "cold" tumors with MHC-I deficiency, but translational challenges require distinguishing MHC-I loss mechanisms.

## Graphical Abstract

**Graphical Abstract:**
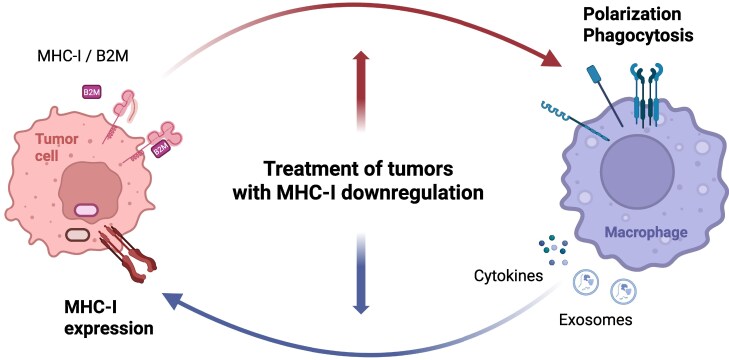

## Introduction

Tumor-associated macrophages (TAMs) are critical components of the tumor microenvironment (TME), exhibiting remarkable plasticity that profoundly influences tumor progression and therapeutic responses [1]. Concurrently, the downregulation of major histocompatibility complex class I (MHC-I) expression on tumor cells, ranging from partial reduction to complete loss, represents a prevalent immune evasion strategy, rendering tumors resistant to conventional T-cell-mediated immunotherapies [2]. While both TAMs and MHC-I downregulation are individually recognized as significant factors in tumor immunity, their intricate bidirectional interplay, particularly in the context of emerging therapeutic strategies for MHC-I-deficient tumors, remains an underexplored yet critical area. This review examines the complex bidirectional regulatory mechanisms between TAMs and tumor MHC-I expression. It also suggests the distinct implications for tumors with reversible versus irreversible MHC-I downregulation, and possible therapeutic strategies targeting TAMs in MHC-I-deficient tumors, highlighting both opportunities and translational challenges.

Initially, macrophages were classified into two polarization states, M1 and M2, based on their opposite enzymatic usage of arginine with inducible nitric oxide synthase (iNOS) and arginase (Arg), respectively [3]. Further studies have unraveled the complexity and diversity of macrophage phenotypes, extending our understanding of their plasticity beyond these two discrete polarization states. For instance, M2 macrophages were further subdivided into M2a, M2b, and M2c subtypes based on the applied stimulation and the repertoire of the produced chemokines [4]. Recently, a novel classification system based on the expression of C-X-C motif chemokine ligand 9 and secreted phosphoprotein 1, for which the term “CS polarity” was coined, has been demonstrated to be superior to the traditional M1/M2 axis with respect to the assessment of clinical outcomes in head and neck squamous cell carcinomas [5]. By mining single-cell transcriptomics data obtained from various cancer types, Ma *et al*. found a generalized TAM transcriptomics pattern, from which they identified seven TAM subtypes common to a variety of cancer types: interferon-primed TAMs, immune regulatory TAMs, inflammatory cytokine-enriched TAMs, lipid-associated TAMs, pro-angiogenic TAMs, resident-tissue macrophage-like TAMs, and proliferating TAMs [6]. Finally, advances in single-cell RNA sequencing and spatial transcriptomics have demonstrated that macrophages in the TME can be characterized not only by their phenotype but also by their interactions with other immune cells and co-localization within the TME. These factors may ultimately define the contribution of TAMs to the antitumor effect [7], and understanding these diverse TAM subsets is therefore critical for developing effective immunotherapies for MHC-I-deficient tumors where conventional T-cell responses are compromised.

To date, immunotherapeutic strategies targeting TAMs primarily involve inhibiting their migration to the tumor nest, depleting them from the TME, or stimulating their direct involvement in the elimination of malignant cells. In particular, interest in reprogramming pro-tumor TAMs to the antitumor phenotype has sharply increased in recent years, although the lack of efficacy remains the main obstacle in translational research [8]. Yet, many macrophage-repolarizing agents are being tested in clinical development, including those targeting the cluster of differentiation 47—signal regulatory protein (SIRP) α phagocytosis axis, stimulator of interferon genes (STING), toll-like receptors, or CD40 costimulatory receptor [8]. However, as the newly described TAM subsets exhibit both antitumor and pro-tumor properties [6], repolarization strategies require careful fine-tuning to suppress their pro-tumor functions while preserving their antitumor functions [8]. Moreover, CellChat analysis mapping interaction networks in the TME identified TAMs as the most interacting cell subpopulation in the TME [9, 10], indicating their central functional role in the TME. Consequently, TAM depletion strategies may not always be beneficial because some TAM-mediated interactions may be required to support antitumor responses.

Downregulation of MHC-I expression, which is responsible for antigen presentation to immune cells, is one of the most common mechanisms used by cancer cells to evade the immune system. In clinical and histopathological settings, the quantification of MHC-I expression on malignant cells is typically performed by immunohistochemical staining and is evaluated relative to healthy surrounding stromal elements or normal infiltrating cells present within the tissue section, which serve as internal positive controls expressing baseline levels of MHC-I molecules [11]. Under normal physiological conditions, MHC-I expression is highly dynamic but strictly coordinated. Healthy cells may possess naturally low or absent MHC-I expression within immune-privileged sites (e.g. the brain, placenta, or testes) [12] or at pluripotent developmental stages (e.g. embryonic stem cells) [13] to prevent autoimmune destruction or maternal-fetal rejection. However, this physiological downregulation is temporary, localized, and readily reversed in somatic tissues by pro-inflammatory signals. In contrast, the downregulation observed in malignant cells is a persistent, pathological process driven by oncogenic transformation and/or selective evolutionary pressure to evade T-cell-mediated surveillance [14, 15].

Tumor cells can silence MHC-I expression either reversibly through epigenetic, transcriptional, and post-transcriptional regulation, or irreversibly through genetic alterations, such as the loss of the β2-microglobulin (B2M) light chain of the MHC-I complex, resulting in either soft or hard lesions, respectively [16]. Conventionally, MHC-I proficient tumors are primarily eliminated by cytotoxic T lymphocytes (CTLs); however, Lerner *et al*. recently demonstrated that CTLs can also target MHC-I deficient tumors, which are predominantly eliminated by natural killer (NK) cells according to the “missing-self” hypothesis [17]. Conversely, MHC-I-proficient tumor cells can be predominantly killed by macrophages even in the presence of specifically activated CD8^+^ T cells [18]. Notably, some therapies, such as modulated electrohyperthermia, can reduce tumor burden while simultaneously decreasing MHC-I expression, leading to a slight decrease in CD8^+^ T cells but a significant increase in the proportion of macrophages, suggesting their important contribution to the antitumor effect [19].

Based on immune cell infiltration patterns, tumors can be categorized as immune-inflamed (or “hot”) and immune-desert or -excluded (collectively termed “cold”). For MHC-I-proficient tumors, which are usually “hot,” immunotherapeutic strategies focus on reactivating CTLs using immune checkpoint inhibitors (ICIs). In contrast, the treatment of “cold” (mostly MHC-I-deficient) tumors remains an immunotherapeutic challenge [20].

Considering the above, and given that macrophages can eliminate malignant cells in an MHC-I-unrestricted manner, antitumor strategies involving TAMs may be particularly beneficial for treating tumors that are poorly infiltrated by CTLs, are inherently “cold,” such as prostate cancer, or where the ICI monotherapy failed to elicit the antitumor response [21]. However, the use of TAMs in immunotherapy for MHC-I-deficient tumors remains largely unexplored. Fortunately, increased awareness of this issue, coupled with improvements in new technologies and the simultaneous rapid development of preclinical tumor models representing tumors with different mechanisms of MHC-I reduction, are believed to be changing this trend. In this review, we aim to critically examine emerging studies on this topic and identify trends in the utilization of TAMs and their role in immunotherapy for MHC-I-deficient tumors.

## TAMs in tumors with irreversibly reduced MHC-I molecules

B2M is a highly conserved, low-molecular-weight protein that is a critical constituent of MHC-I. Being noncovalently bound to the α heavy chain of MHC-I, B2M ensures the formation of a functional and stable peptide-MHC-I complex. In cancer cells, genetic inactivation of B2M results in the loss of MHC-I, impairing the cell’s ability to present antigens to CD8^+^ T cells. Consequently, this deficiency hampers CD8^+^ T-cell-mediated elimination of malignant cells *via* the T-cell receptor. Therefore, B2M-deficient tumors are usually classified as “cold” tumors [22], creating a bottleneck for CTL-based immunotherapy approaches.

To address this limitation, new and alternative immune pathways as well as therapeutic approaches need to be explored using relevant preclinical tumor models. In recent years, knocking out the *B2m* gene in cancer cell lines has been widely applied to study the effects of this molecule on tumors and the TME in both *in vitro* and *in vivo* settings. First, *in vitro* genetic removal of B2M using clustered regularly interspaced short palindromic repeats (CRISPR) and CRISPR-associated protein (Cas) 9 technology has been shown to differentially affect cell properties. For instance, murine colorectal cancer cell lines CT26 and MC28, which were devoid of functional *B2m*, proliferated comparably to the parental cells, but tumors induced with these cell lines progressed significantly faster than those induced with parental cell lines [23]. In contrast, knocking out *B2m* in human papillomavirus-associated TC-1 cells [24] and breast cancer 4T1 cells [10] resulted in significantly reduced proliferation rates and decreased tumor growth compared to their parental counterparts. The melanoma B16-F10 and breast cancer EO-771 clones with a deactivated *B2m* gene formed tumors in mice only after NK cell depletion [25]. Li *et al*. knocked down *B2M* in the human-derived glioblastoma stem cells (GSC) and observed decreased cell proliferation, viability, and self-renewal *in vitro* [26]*. In vivo*, brain tumors induced with these cells exhibited delayed tumor manifestation and reduced tumor volume. These findings suggest that genetic manipulations aimed at reducing B2M levels in preclinical models affect individual cell lines differently. This cell-line-dependent variability in tumor progression following B2M knockout indicates that the resulting TME, including TAM composition and function, may also differ significantly, necessitating careful model selection for TAM-focused studies.

Few studies have examined the effects of *B2M* knockdown on the phenotypes and functions of TAMs in the TME of individual cancer types. Li *et al*. conducted a series of *in vitro* experiments in which they induced M1 and M2 macrophage phenotypes from the THP-1 human monocyte cell line, and these macrophages were subsequently exposed to conditioned medium from B2M-silenced human-derived GSCs [26]. Next, an *in vivo* investigation was performed using intracranial mouse xenografts obtained by implantation of these B2M-silenced GSCs. Compared to unmodified GSCs, in both *in vitro* and *in vivo* experiments, M2 markers on TAMs were significantly decreased, while M1 TAM markers remained unchanged, indicating that B2M produced by tumor cells supports the pro-tumor properties of TAMs in GBM. In multiple myeloma (MM), increased levels of B2M secreted by malignant cells can be internalized by macrophages, leading to pro-inflammatory responses by activating the NLR family pyrin domain containing 3 inflammasome and subsequent production of pro-inflammatory cytokines, such as interleukin (IL)-1β and IL-18. These cytokines, in turn, support MM progression [27], establishing TAMs as key promoters of MM development. Taken together, B2M enhanced the pro-tumor characteristics of TAMs in GSC and MM tumors.

In the clinical landscape of colorectal cancer, B2M-deficient tumors are often classified as “cold” and are generally associated with poor prognosis [28, 29]. These tumors exhibited reduced infiltration of CD8^+^ T cells and CD163^+^ macrophages, correlating with lower disease-specific survival [29]. Gurjao *et al*. identified the loss of the *B2M* gene as an intrinsic resistance mechanism in a metastatic colorectal cancer patient who experienced disease progression after ICI treatment, despite having a high neoantigen load and the presence of prognostic markers such as tumor DNA mismatch repair deficiency and high microsatellite instability [30]. These markers are typically associated with better responses to ICI-based immunotherapy. Notably, this patient exhibited the highest infiltration of activated NK cells and M2 macrophages compared to multiple colorectal cancer cases in the TCGA database. In conclusion, B2M-deficient colorectal tumors are characterized by an unresponsive immune landscape, including altered TAM populations with a predominance of immunosuppressive M2 macrophages.

In addition to its role as a stabilization unit in the MHC-I complex, B2M expressed on tumor cells can function as a “don’t eat me” signal for phagocytes by binding to the inhibitory receptor leukocyte immunoglobulin-like receptor subfamily B 1 (LILRB1) on their surface. The MHC-I/(B2M)-LILRB1 axis constitutes an anti-phagocytic signal, enabling cancer cells to evade clearance by macrophage-mediated phagocytosis [31]. Therapeutic interventions using monoclonal antibodies to block LILRB1 have emerged as a potent strategy to disrupt the MHC-I/(B2M)-LILRB1 inhibitory axis (Table 1), effectively restoring macrophage activation and promoting the engulfment of wild-type malignant clones [32]. This pathway functions in close coordination with the canonical CD47-SIRPα “don’t eat me” axis [32, 33]. Because genetic disruption or loss of *B2M* prevents the MHC-I complex formation and surface expression, tumor cells lose their primary LILRB1-mediated defense. Consequently, the CD47-SIRPα signaling becomes the dominant protection mechanism against innate clearance. This makes B2M-deficient tumor cells particularly vulnerable to phagocytosis and significantly more responsive to anti-CD47 monotherapies than their wild-type counterparts [32].

**Table 1:** Therapeutic strategies targeting macrophage functions in tumors with dysregulated MHC-I expression.

Therapeutic strategy	Representative agent(s)	Primary mechanism of action	Effect on tumor microenvironment
LILRB1 blockade	Monoclonal antibodies against LILRB1	Disruption of the inhibitory MHC-I/B2M-LILRB1 “don’t eat me” signaling cascade	Restores macrophage activation; promotes phagocytosis of MHC-I-proficient malignant clones
CD47-SIRPα checkpoint inhibition	Monoclonal antibodies against CD47	Disruption of the inhibitory CD47-SIRPα “don’t eat me” signaling cascade	Induces robust TAM-mediated engulfment of unshielded, B2M-deficient tumor cells
cIAP1/2 antagonism	LCL161 inhibitor	Induction of apoptosis by elimination of cIAP1/2	Drives accumulation of highly phagocytic $\mathrm{Ly}6C^{+}\mathrm{MHC}\text{-}II^{+}$ TAMs; prompts tumor cells to release pro-inflammatory cytokines
Cytokine mRNA delivery + targeted mAbs	Encapsulated mRNA encoding ILs (IL-2, IL-12) + tumor-targeting mAbs/anti-PD-L1	Co-delivery of encapsulated cytokine mRNA alongside opsonizing or checkpoint-targeting antibodies	Drives polarization into $\mathrm{MHC}\text{-}II^{+}$ M1 TAMs; enhances antibody-dependent cellular phagocytosis and antigen cross-presentation to $\mathrm{CD}8^{+}$ T cells
TGF-β receptor inhibition	Galunisertib (LY2157299)	Small-molecule inhibition of the TGF-β type I receptor kinase domain	Arrests downstream SMAD signaling; prevents B2M/PIP5K1A-induced M2-like macrophage polarization
TGF-β trapping and neutralization	Bintrafusp alfa/ligand-neutralizing antibodies	Inhibition of the TGF-β signaling pathway	Depletes active TGF-β from the TME; disrupts the hyperactive cytokine axis to suppress tolerogenic TAM development
Multi-kinase inhibition	Q702 inhibitor	Selective inhibition of Axl, Mer, and CSF1R kinases on both tumor and immune cells	Concurrently upregulates surface MHC-I on tumor cells and reprograms M2 phenotypes into M1 TAMs
NK-derived extracellular vesicles (NEV)	NEV conjugated with engineered CTLs	pH-triggered release of tumor-homing NEVs from CTLs to induce tumor cell apoptosis	Upregulates MHC-I on tumor cells; significantly increases the M1/M2 macrophage ratio and $\mathrm{CD}8^{+}$ T cell infiltration
Peptide-drug conjugates	Sudocetaxel Zendusortide (TH1902)	Docetaxel conjugated to a sortilin 1-targeting peptide TH19P01	Triggers the intracellular cGAS/STING pathway; elevates tumor MHC-I expression and increases total infiltration of immune cells
PP2Ac inhibition	LB-100 inhibitor	Inhibition of PP2Ac catalytic subunit	Restores/upregulates tumor MHC-I expression; activates the STING-controlled type I IFN pathway and M1 repolarization
Exosome-mediated immunomodulation	M1-type exosomes loaded with celastrol	Induction of tumor apoptosis and enhancement of TAM phagocytosis	Downregulates MHC-I on tumor cells; suppresses M2 TAMs while promoting M1 polarization

This increased susceptibility to macrophage-mediated engulfment provides a strong rationale for exploiting phagocytosis as a therapeutic strategy. Furthermore, some studies have highlighted the phagocytic potential of macrophages as a therapeutic option for B2M-deficient tumors. For instance, Xi *et al*. demonstrated that nonpolarized RAW 264.7 macrophages exhibited enhanced phagocytosis of B2M-deficient tumor cells compared to wild-type cells [10]. In B2M-deleted colorectal MC38 cells and pancreatic 6694c cells, the application of the cellular inhibitor of apoptosis (cIAP)1/2 antagonist LCL161 resulted in the accumulation of Ly6C^+^ MHC-II^+^ TAMs with increased phagocytic capabilities toward live tumor cells [21]. A similar observation was made by Zhang *et al*. in 4T1-induced tumors with a deactivated *H2-K1* gene, where treatment with nanoparticles loaded with LCL161 and decorated with macrophage membranes naturally expressing SIRP-α facilitated the interaction with CD47 on tumor cells, allowing the internalization of nanoparticles by cancer cells [34]. The treated tumor cells produced pro-inflammatory cytokines, including granulocyte-macrophage colony-stimulating factor, monocyte chemoattractant protein-1, IL-8, tumor necrosis factor (TNF)-α, and type I interferons (IFN), which in turn polarized macrophages toward a phagocytic subtype defined as Ly6C^+^ MHC-II ^+^ . LCL161-nanoparticle-based treatment also inhibited tumor growth and progression in models resistant to anti-programmed cell death protein 1 (PD-1)/PD-1 ligand 1 (PD-L1) immunotherapy. However, in both studies [21, 34], the antitumor effect was not solely attributed to TAM-mediated phagocytosis. Although direct cytotoxicity by CTLs was probably not possible due to MHC-I deficiency, CTLs were still indispensable for the antitumor effect as a source of lymphotoxin.

Similarly, IL-based immunotherapies can also rely on TAM-mediated support in CTL-driven antitumor responses in B2M-deficient tumors. Two recent studies reported pronounced TME remodeling accompanied by repolarization of TAMs as a result of combined treatment with encapsulated mRNA encoding ILs and tumor-targeting monoclonal antibodies (mAbs) [23, 35]. Specifically, in B2M-deficient colorectal MC38 and melanoma B16-F10 tumor models, stably expressing Her2 and Trp1 tumor-specific antigens, respectively, it has been demonstrated that co-delivery of IL-2 mRNA with the corresponding mAb resulted in a marked increase in MHC-II^+^ M1 TAMs, high CTL infiltration, and durable tumor control [23]. Similarly, application of IL-12 mRNA in combination with anti-PD-L1 in B2M-negative YUMMER1.7- and MC38-induced tumors enhanced proportions of M1 TAMs and sustained antitumor immunity [35]. Mechanistically, TAMs contributed to the antitumor response by engulfing mAb-opsonized tumor cells, which mediated antibody-dependent cellular phagocytosis (ADCP) and cross-presentation of tumor antigens to CD8^+^ T cells [23]. Collectively, these findings suggest that the ability of TAMs to phagocytose tumor cells, either directly or indirectly through ADCP, provides a potential therapeutic strategy for B2M-deficient tumors. However, the effectiveness of this approach and the overall antitumor effect remain T-cell-dependent.

In cells lacking B2M, MHC-I heavy chains can still be expressed on the cell surface as open conformers [36]. Such molecules have been found on both mouse and human B2M-deficient cells [37–40] and their expression could be increased under inflammatory conditions [24, 41, 42]. Open MHC-I conformers can interact in *cis* or *trans*, forming homodimers or oligomers, or binding receptors for growth factors, cytokines, hormones, and neurotransmitters, thereby modulating their downstream signaling [36, 43], but the effect of open conformers on TAMs has not yet been reported.

**Figure 1:**
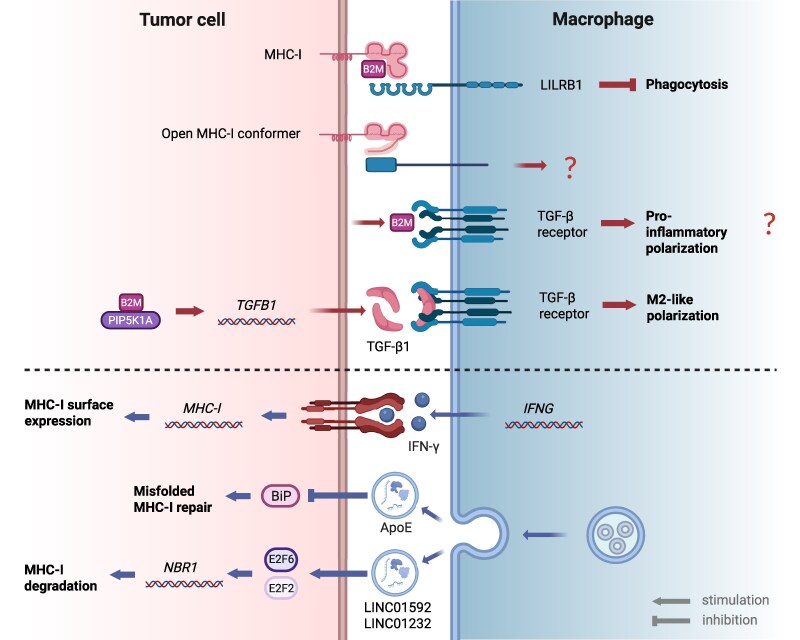
Relationship between macrophages and MHC class I molecules in tumor cells. The antitumor activity of TAMs may be influenced by MHC-I expression in tumor cells (upper part), and conversely, MHC-I expression in tumor cells may be increased or decreased by TAM products released in the TME (lower part). As indicated by the question marks, some possible effects have not yet been verified for TAMs. ApoE, apolipoprotein E; B2M, beta-2-microglobulin; BiP, binding immunoglobulin protein; E2F2(6), E2F transcription factor 2(6); IFNG, interferon gamma; LILRB1, leukocyte immunoglobulin-like receptor B1; LINC, long intergenic noncoding RNA; MHC-I, MHC class I; NBR1, neighbor of BRCA1 gene 1; PIP5K1A, phosphatidylinositol-4-phosphate 5-kinase type 1 alpha; TAM, tumor-associated macrophage; TGFB1, transforming growth factor beta 1; TME, tumor microenvironment. Figure 1 was created in BioRender. Smahel, M. (2026) https://BioRender.com/dv5s0oc.

The total loss of surface MHC-I molecules also arises from the inactivation of genes encoding MHC-I heavy chains [44]. This mechanism of MHC-I deficiency might also affect the TME, including the polarization and activity of TAMs. Besides missing MHC-I heavy chains, B2M may play a role in this process (Fig. 1), because B2M can function not only as an integral component of MHC-I that enables antigen peptide presentation and inhibits macrophage activity through interaction with LILRB1 [32], but also in a soluble form as a growth factor affecting tumor growth and progression [45]. Furthermore, circulating B2M has been demonstrated to induce a pro-inflammatory phenotype in blood monocytes by binding to the transforming growth factor β (TGF-β) receptor and activating noncanonical SMAD-independent signaling [46]. Although the influence of B2M on TAMs polarization *via* the TGF-β receptor has not been described yet, TGF-β induced by B2M binding to phosphatidylinositol-4-phosphate 5-kinase type 1 alpha (PIP5K1A) was reported to promote M2-like polarization [26]. Several strategies targeting the TGF-β pathway are being evaluated to break this immunosuppressive loop [47]. One advanced approach utilizes small-molecule inhibitors of the TGF-β type I receptor (TGF-β RI/ALK5), such as galunisertib, which block downstream SMAD signaling and successfully prevent M2-like macrophage polarization [48, 49]. Alternatively, ligand-neutralizing antibodies or bifunctional fusion proteins that trap active TGF-β (such as bintrafusp alfa) are used to deplete this cytokine from the TME [50]. These therapeutic interventions suppress the development of tolerogenic TAMs and significantly enhance macrophage-mediated tumor clearance.

## TAMs in tumors with reversibly reduced MHC-I molecules

The reversible downregulation of MHC-I molecules is mostly caused by epigenetic silencing of components of the antigen processing and presenting machinery (APM), which can be reversed by treatment with cytokines, such as IFN-γ or TNF-α. This silencing specifically results from promoter hypermethylation or histone modifications of APM genes [16]. In addition, the impaired expression or activation of essential transactivators, such as the NLR family CARD domain containing 5, the master transcriptional coactivator of MHC-I genes; nuclear factor kappa B (NF-κB); and interferon regulatory factor 1 directly halts the transcriptional initiation of the antigen-presentation pathway [15]. This transcriptional block is often regulated by cell-intrinsic oncogenic signaling pathways, such as the MYC proto-oncogene, the epidermal growth factor receptor, and the mitogen-activated protein kinase/extracellular signal-regulated kinase pathways, which suppress these transcription factors (TFs) through transcriptional and post-transcriptional mechanisms to facilitate immune evasion [15, 51]. This immunosuppressive network is further exacerbated by extrinsic TME stressors, such as hypoxia-induced hypoxia-inducible factor 1-alpha signaling, which also impairs antigen presentation [52]. Consequently, restoring the antigen presentation capability of tumor cells is a critical step in successful immunotherapy. Moreover, an increase in surface MHC-I expression on tumor cells can often be accompanied by further changes in the TME, such as the reprogramming of macrophages from the M2 to M1 phenotype. For instance, treatment of tumors induced with EMT6 cells, representing triple-negative breast cell carcinoma, with the Q702 inhibitor of Axl, Mer, and CSF1R kinases targeting both tumor and immune cells resulted in significant tumor volume reduction accompanied by an increase in MHC-I on tumor cells and repolarization of M2 to M1 TAMs despite the overall decline in TAMs subpopulation [53]. In another example [54], a pH-sensitive two-component system, based on chemically modified NK-derived extracellular vesicles (NEV) conjugated with chemically engineered CTLs, was applied to treat B16-OVA-induced tumors, which are known for their inherent decrease in MHC-I molecules [55]. The CTL-NEV formulation significantly increased MHC-I expression, M1/M2 macrophage ratio, and proportion of CD8^+^ T cells compared to the untreated group. This was accompanied by tumor growth inhibition and longer survival of mice compared to treatment with NEV and CTL monotherapies or the untreated group. These findings suggest that M1 macrophages (CD80^+^ CD86^+^) and CD8^+^ T cells contributed to the antitumor effect.

In contrast to the relatively clear concept of the genetic elimination of MHC-I components, such as the B2M light chain, resulting in irreversible MHC-I downregulation, the derivation of cell clones with reversible MHC-I downregulation remains poorly described. Consequently, there is a limited number of reports on the development of relevant preclinical models and corresponding studies investigating the TME of such tumors for its modulation by immunotherapy. The TC-1/A9 model is one of the few intentionally developed preclinical models of reversible MHC-I downregulation. It is a clone derived from TC-1 cells that express the HPV16 oncoproteins E6 and E7. This model is characterized by reduced *Tap1* expression, which accounts for the decreased surface MHC-I expression [56]. TC-1/A9-induced tumors are resistant to anti-PD-L1 monotherapy but responsive to IFN-γ treatment [57]. It has been demonstrated that immunotherapy increased MHC-I expression on tumor cells and transformed the TME from “cold” to “hot” [57, 58]. This change was accompanied by collaboration between CD8^+^ T cells and NK cells, as well as the reprogramming of M2 macrophages into M1 macrophages. In addition, the antitumor effect in both studies was enhanced by the depletion of CD4^+^ cells. In the transient antitumor effect achieved by the combined immunotherapy with DNA immunization and immunostimulatory CpG motifs (ODN1826) administration, TAMs from TC-1/A9-induced tumors exhibited an M1 phenotype, as evidenced by increased levels of MHC-II molecules and iNOS expression [58]. Notably, by the usage of key immune response markers that were upregulated during immunotherapy in TC-1/A9 tumors, a human-reflective genetic signature was established. Patients with increased expression of this genetic pattern showed significantly improved overall survival [57]. This finding supports the TC-1/A9 model as a relevant preclinical model for translational research.

A major challenge in cancer immunotherapy is converting “cold” tumors into “hot” tumors. Increasing the surface expression of MHC-I molecules on tumor cells is a critical step in this process. Several studies have investigated strategies to induce this switch in preclinical tumor models with reversible MHC-I expression, including B16-F10 melanoma and SB28 glioblastoma tumors. In the B16-F10 melanoma model, Demeule *et al*. applied Sudocetaxel Zendusortide (TH1902), a peptide-drug conjugate, which combines a chemotherapeutic agent docetaxel conjugated to the TH19P01 peptide that binds to sortilin 1, a receptor that is highly expressed on cancer cells and mediates ligand internalization [59]. TH1902 treatment produced sustained antitumor effects, as evidenced by significant tumor growth inhibition accompanied by a marked increase in surface MHC-I expression and robust infiltration of CD45^+^ immune cells compared to vehicle- or docetaxel-treated tumors. The infiltrating lymphocytes included CD161c^+^ NK cells, CD8^+^ CTLs, CD4^+^ T helper cells, and FoxP3^+^ regulatory T cells. TAM infiltration also increased, with comparable levels of both CD68^+^ M1 and CD206^+^ M2 macrophage subtypes. These findings indicate that while reprogramming the TME from “cold” to “hot” can be achieved, fine-tuning the balance and phenotype of immune infiltrates remains crucial for optimizing therapeutic outcomes.

In another study, Mondal *et al*. investigated the impact of protein phosphatase 2 catalytic subunit alpha (PP2Ac) deficiency in the SB28 glioblastoma model, which resembles the immunologically “cold” features of human glioma, such as low mutational burden, reduced MHC-I expression, and resistance to ICB therapy [60]. Using CRISPR-Cas9 to remove the PP2Ac subunit, the authors observed a notable increase in surface MHC-I expression compared with wild-type tumors, a finding confirmed across a few different brain tumor models. Single-cell RNA sequencing revealed substantial remodeling of the myeloid cell population in PP2Ac-deficient tumors. Genes associated with M2-like macrophages, such as *Mrc1* (*CD206*) and *Arg1*, were downregulated, whereas TAM clusters with increased type I/II IFN signature, NF-κB activation, and antigen processing/presentation were enriched. In contrast, wild-type tumors displayed an enrichment of TAM clusters characterized by oxidative phosphorylation metabolism and a low IFN signature, which is typical of immunosuppressive M2-like phenotypes. These findings underscore the importance of identifying factors such as PP2Ac, whose disruption can unlock the potential of TME to be remodeled from “cold” to “hot” which can be accompanied by TAM repolarization and MHC-I upregulation. Importantly, this genetic vulnerability can be exploited therapeutically through the pharmacological inhibition of PP2Ac using small-molecule inhibitors such as LB-100. This cell-permeable compound phenocopies the genetic deletion of PP2Ac by selectively binding to its catalytic domain, thereby arresting DNA damage repair pathways, triggering the STING-controlled type I interferon axis, and successfully driving the repolarization of the myeloid stroma to overcome resistance to traditional immune checkpoint blockade [60, 61].

## TAMs’ interactions with tumor MHC-I molecules

Another rationale for targeting TAMs in tumors with downregulated MHC-I expression is their ability to modulate tumor immunogenicity through various mechanisms. Under certain immunotherapeutic conditions, TAMs can upregulate MHC-I expression on cancer cells through IFN-γ production (Fig. 1). In the B16 melanoma model, which is MHC-I-negative *in vitro* but weakly positive *in vivo* [55], it has been demonstrated that IL-2-transduced B16 cells activated TAMs to produce high levels of IFN-γ, leading to the induction of MHC-I expression in cancer cells. Conversely, depletion of the TAM subpopulation impaired MHC-I upregulation. These findings indicate that TAMs can restore MHC-I expression on tumor cells when exposed to favorable microenvironmental cues [55].

In contrast, M2 TAMs can suppress MHC-I-dependent antigen presentation in tumor cells *via* exosome-mediated mechanisms. For instance, apolipoprotein E, transmitted by M2 TAM-derived exosomes, reduces tumor immunogenicity by interfering with the ATPase activity of the binding immunoglobulin protein (BiP) in the endoplasmic reticulum (ER) [62]. This inhibition prevents BiP from repairing misfolded MHC-I, thereby impairing peptide loading onto the MHC-I complexes. Consequently, antigen presentation is compromised in the ER, rendering tumor cells with reduced surface MHC-I expression invisible to CTLs.

Recent studies have identified additional exosome-mediated mechanisms by which TAMs regulate MHC-I expression in tumors (Fig. 1). In esophageal cancer (EC) [63] and glioma [64] models, M2 TAM-derived exosomes transfer the long noncoding RNAs LINC01592 and LINC01232, respectively. These RNAs bind to the TFs E2F6 (in EC) and E2F2 (in glioma), promoting their nuclear translocation and subsequent activation of neighbor of BRCA1 gene 1 (*NBR1*) transcription. The autophagy cargo receptor NBR1 binds to ubiquitinated MHC-I and facilitates its degradation in autophagolysosomes. This reduction in surface MHC-I expression enables tumor cells to evade CTL-mediated killing. Importantly, silencing LINC01592 or LINC01232 restored MHC-I expression and resensitized cancer cells to immune attack. Consistently, analyses of clinical tumor samples revealed an inverse correlation between lnRNAs/TFs/NBR1 and MHC-I levels, particularly in high-grade cancers. Collectively, these findings highlight that TAM-derived exosomes can suppress MHC-I expression in tumors, promoting immune evasion.

Exosomes are increasingly being used for the development of therapeutic products. Taking advantage of macrophage-derived exosomes to modulate tumor immunogenicity, Yu *et al*. developed an M1-type exosome system derived from IFN-γ-stimulated RAW 264.7 macrophages and loaded with the bioactive compound celastrol for the treatment of tumors induced with MDA-MB-213 cells, a model of triple-negative breast cancer [65]. This platform exhibited pleiotropic antitumor activity: it enhanced the phagocytic capacity of TAMs *in vitro*, reduced MHC-I expression on tumor cells by inhibiting the interferon regulatory factor 1-HLA class II transactivator signaling pathway, decreased the proportion of CD163^+^ M2 TAMs, and promoted the polarization of CD80^+^ M1 TAMs. The potent antitumor effect, mediated by phagocytic TAMs, was thus induced by both TAM polarization and MHC-I downregulation in tumor cells.

## Conclusion and perspectives

This review highlighted the multifaceted and bidirectional interactions between TAMs and MHC-I molecules derived from tumor cells, emphasizing their critical roles in immune evasion and therapeutic response in both irreversibly and reversibly MHC-I-deficient tumors. The mechanisms underlying this communication are still only partially understood. However, emerging sophisticated methodologies hold promise for deeper clarification. Specifically, recent advances in single-cell sequencing and spatial transcriptomics and proteomics provide unprecedented opportunities to dissect the TME at a high resolution and uncover cellular interactions that were previously overlooked by bulk or low-multiplex analyses. These advanced approaches can particularly provide insights into how macrophage-mediated mechanisms contribute to antitumor immunity or drive pro-tumor activities, ultimately remodeling the TME, as already demonstrated in several experimental models [66–69]. Therapeutic interventions against tumors with downregulated MHC-I expression should be tailored to the cause of the downregulation. For hard lesions with irreversible MHC-I deficiency mediated by B2M loss, therapies should focus on promoting macrophage phagocytosis by blocking the CD47-SIRPα pathway, inhibiting cIAP1/2, or delivering cytokine-expressing mRNA. Tumor-targeting antibodies should also be administered to enhance ADCP. Conversely, tumors with preserved *B2M* expression could specifically benefit from TGF-β signaling inhibition. Therapeutic strategies for reversible soft lesions should simultaneously restore tumor MHC-I expression and repolarize TAMs to the antitumor phenotype, such as multi-kinase inhibition with Q702, PP2Ac antagonism, or targeted docetaxel delivery. Future progress also depends on modifying exosomes to enhance their therapeutic potential.

In conclusion, advanced single-cell and spatial analyses offer novel insights into the role of TAMs in the TME of MHC-I-deficient tumors and their therapeutic potential. Nonetheless, a critical need remains for the development of diverse preclinical models accurately reflecting different mechanisms of MHC-I reduction to study the interactions of TAMs with other cells in the TME more precisely. Preclinical therapeutic models demonstrate that tumor MHC-I loss enhances TAM phagocytosis while disrupting pro-tumor polarization. Although macrophage-mediated phagocytosis of tumor cells is a key antitumor mechanism, which can be leveraged in cancer immunotherapy, the overall therapeutic efficacy of TAM-targeted strategies frequently relies on and is enhanced by synergy with CD8^+^ T-cell responses. In addition, new therapeutic targets are being identified through single-cell analyses of the TME. These observations highlight both the promise and limitations of harnessing TAMs in the immunotherapy of MHC-I-deficient tumors. However, translational challenges persist, driven by the need for tailored TAM strategies to address diverse MHC-I defects. Ultimately, the future of treating immunologically cold tumors with MHC-I downregulation lies in integrating personalized diagnostic profiling of MHC-I defects with therapeutic regimens that coordinate the stimulation of macrophage phagocytosis and adaptive T-cell responses.

## Data Availability

Not applicable.

## Permission to reproduce

The graphical abstract was created in BioRender. Smahel, M. (2026) https://BioRender.com/0j34633.

Figure 1 was created in BioRender. Smahel, M. (2026) https://BioRender.com/dv5s0oc
